# Some Remarks on Small-Pox, Varioloid and Varicella, as Connected with Their Diagnosis

**Published:** 1853-10

**Authors:** 


*Some Remarks on Small-pox, Varioloid and Varicella, as connected with their Diagnosis.* By Charles A. Lee, M. D., Professor of Materia Medica and Pathology, in Geneva Medical College, etc., etc.

In the July number of the *American Journal of the Medical Sciences*, (1853,) I gave some account of a varioloid disease, which has recently prevailed to a considerable extent, in the townships of Gorham and Phelps, Ontario county, state of New York. In that article I gave a pretty minute description, from careful personal observation of the phenomena; particularly the character of the eruption, in different cases, showing that, in many instances, the eruption bore all the distinguishing marks of varicella, as well as purpura, pemphigus, pompholyx, and even erysipelas; so that some experienced physicians were so confident that the disease was *chicken-pox*, that they staked their professional reputation on the issue, and refused to advise vaccination, or re-vaccination, in cases where persons were liable to exposure. Even after well-marked cases of *small-pox* appeared, which were distinctly traceable to the same infection, (and all the cases could be traced to the same source,) some still persisted that the previous cases were *chicken-pox*, and that *these* were only more aggravated cases of the same disease.

From what I have observed, I am inclined to believe that this mistake is constantly being made, at least in our cities, and that is one reason why small-pox is an endemic in such places; the virus being constantly disseminated in some of its milder forms of *varioloid*, which are mistaken for *varicella*. I have myself known this mistake made in repeated instances; and there is the greater danger of making it in large cities, inasmuch as it is often impossible to trace the infection, as in the country, to its original source. One medical gentleman, of Gorham, a most respectable practitioner of some forty years' standing, who has treated many cases of small-pox, and had charge of a small-pox hospital, still insists, I believe, that the disease of which I gave some account, was *varicella*, although, he admits, if I am not mistaken, that small-pox prevailed at the same time. But inasmuch as every case could be traced directly to a Mr. McComb, who brought the disease from Rochester, they must all be regarded as the same disease, although their distinctive characters, in different cases, were exceedingly variant:—a somewhat further consideration of these diseases, may, perhaps, be useful.

Hufeland describes chicken-pox under the name of "*variola spuria*," and remarks, under the head of diagnosis, that "the pustules are sometimes small and *perfectly similar to small-pox*, sometimes found only on single parts, and sometimes everywhere. They break out after a febrile commotion of twenty-four hours, which is, in some cases, very slight and scarcely perceptible, and *in others violent*, and increases to delirium. Some pustules form within the first twenty-four hours, and dry up in twenty-four hours more; but a few single pustules will continue to suppurate for a longer time." "The distinction," continues Hufeland, "between this and genuine small-pox, therefore, consists *neither in the form*, nor in the fever, which sometimes is very slight in the genuine and violent in the spurious, according to variety of constitution; but merely in the quickness of its course. In the genuine variolous disease, there are three days of fever before the eruption; in the spurious, only one day; in the genuine there are three days for suppuration; in the spurious, one day; the same proportion for exsiccation, so that the genuine small-pox completes its course within nine or twelve days — the spurious, within three or four days. Life is never endangered by this disease."* The above marks are too often lost sight of; had they been noticed, the disease would probably not have been mistaken for varicella. In no case did the eruption appear on the second day, so far as I could learn, and when it did appear, it came out generally in successive crops, assuming a great variety of forms: as *varicella*, *pemphigus*, *small-pox*, *purpura*, *swine-pock*, *horn-pock*, &c., &c. There is much truth in the remark of Dr. Mason Good, that, "perhaps there is no exanthem that is so much affected by accidental influences as the small-pox;" and he further observes, that these "diversities, or aberrations of small-pox, more commonly approximate the form and general character of pemphigus, or varicella, (chicken-pox,) and have, no doubt, often been mistaken one for the other, especially the latter, of which the severe variolous epidemics, that have of late years spread over Edinburgh, and other parts of Scotland, as well as over many parts of the Continent, afford striking examples,"† &c.

An anomaly was observed in several instances in the disease, as it prevailed at Gorham, which served still more to render the diagnosis difficult—and that was, individuals who had not been vaccinated, *had the disease* in a very mild form; indeed, no one could distinguish them from varicella. This, however, has been noticed in similar epidemics. Thus Dr. Mallock, in a

---

* Prac. of Medicine. By C. W. HUFELAND. Am. Ed., p. 395.
† Study of Medicine, vol. 1, p. 625, Am. Ed.

letter to Dr. Thomson, of Edinburgh, makes the following statement:—"No case of small-pox had occurred in this town for nine years, till last winter, when an idle boy, who was in the habit of wandering about the country, frequenting markets, &c., happened to be at a house, where some of the inmates were said to be ill of small-pox. He himself had been vaccinated some years before. On his return home he was seized with febrile symptoms, and confined for two or three days in bed, *when an eruption similar to chicken-pox* made its appearance. Immediately the fever abated, and in a few days more he left his bed, and attended a cattle-market, half a mile distant from the town, without experiencing any bad consequences. About a week afterward, one of his master's children was taken ill, and went through the regular stages of small-pox in a mild manner; then a second similarly; a third suffered in a very alarming degree, from the confluent kind; a fourth rather worse than the two first, and the youngest, of eight months old, had what, if the other cases had not occurred, *I would, without hesitation, have called chicken-pox;* for there was little or no fever, and the pustules were filled with a watery fluid, which was not converted into the purulent appearance of small-pox. None of these children had undergone vaccination."*

The same thing occurred repeatedly in Gorham, even in the same family; some, who had not been vaccinated, having the disease in the mild varicelloid form, while others, who had been vaccinated, had a pretty severe varioloid attack. But as a general rule, those who had been vaccinated, had a milder form of the disease than those who had not. It is a very great mistake to suppose that in all cases of small-pox, or varioloid, the pustules must contain pus, or even a milky, sero-purulent fluid. They may, as in varicella, contain limpid serum, and differ in no respect from the eruption of chicken-pox. In perhaps one-half the cases, the fluid was thin, and whey-like in aspect, whether the fever and constitutional symptoms were mild or severe. Sometimes the eruption was at first *papular*, then became *vesicular;* either conical, or having a depressed point in the center, with pellucid lymph in the early stages, gradually becoming milky, and when matured, containing the same whey-like matter, but rarely assuming that thick consistence and yellow color, which usually occurs in true small-pox, and this, too, whether preceded by vaccination or not. The number of vesicles varied from five or six, or even none at all, although constitutional symptoms were present. It would be wrong, however, to infer, that the controlling power of the vaccine disease was not exhibited in this epidemic, for, as a general rule, the eruption was of a more varicelloid form, and the constitutional symptoms much milder in cases where vaccination had previously been performed; though there were some striking and remarkable exceptions to this. For the most part appearing on the third, they reached their size by the fourth day after their appearance, and were speedily followed by desquamation, to be succeeded by other crops, some of which were drying and scaling off, as others were making their appearance.

There were instances, too, where, as in cases described by Thomson and Marshall,† the skin assumed a measly appearance, the surface of the body resembling wet brown, or wrapping paper, and in one instance, where the

---

* Thomson, Variol. Epidem., p. 333.

† *Account of the Introduction of Vaccination,* &c., in Ceylon, &c. (1819.) By HENRY MARSHALL, M. D.

eruption was confluent, Drs. Potter and Dean were inclined to regard the case, which proved fatal, as one of erysipelas; the whole body presenting a bright efflorescence, but without any effused fluid. In other cases, the vesicles contained air, and in one case, blood was extensively effused in spots and patches beneath the entire lower extremities. This case recovered, and what was remarkable, there was but slight fever, or constitutional disturbance; abscesses, too, formed in some instances, toward the close of the disease, filled with bloody ichor or pus seated on different parts of the body, and which proved exceedingly obstinate and annoying. Every one acquainted with the history of small-pox knows that the different forms which the eruption assumes have led to many controversies; the measles, plague and chicken-pox, &c., all having been believed to be varieties of small-pox. This controversy, with regard to the identity of chicken-pox and small-pox, which had raged for several centuries, appeared to be almost settled in the negative, when the work of Professor Thomson, of Edinburgh, appeared in 1820, to revive the dispute, and furnish still further evidence in favor of the identity of the two diseases; and what served still further to confound them, the lymphatic small-pox was called chicken-pox, to distinguish it from the purulent variety, by Harvey, Mead, Morton, and others; while *varicella* or *water-pox*, in all its forms, was called *variola*, though the term *spurious* or *bastard*, was generally prefixed. Professor Frank, about half a century since, undertook to show that *chicken-pox, horn-pox*, and all those diseases, which have been called *varioloid* diseases, as well as *pompholyx*, belong to *pemphigus*, as a genus, of which there are two species, *amplior* and *variolodes*. Heberden and Willan entertained more correct notions, and tried to discriminate between the two diseases, which they did to the satisfaction of the great body of the profession, down to the publication of Professor Thomson's work in 1820, and even now, in this country, few, perhaps, can be found who would contend for the identity of *variola* and *varicella*. The phenomena I have described, as connected with the varioloid epidemic at Gorham, have been observed in other instances, as in Scotland, for a series of years, as described by Thomson* and others; also at Montpelier, in France, in 1816, where, according to Professor Berard, the eruption presented all the forms which were observed at Gorham, whether as respects the shape and number of pustules, the nature of their fluid, the length of time required for exsiccation and desquamation, the duration and severity of the eruption, as well as in the absence or presence of the secondary fever. It was the opinion of some physicians at Gorham, as at Montpelier, that the chicken-pox preceded and accompanied the genuine variola; while the two were often so blended, that it was difficult, if not impossible, to draw the line of demarkation. I might, with propriety, use the same language in reference to the disease, as Berard employs: "*Never, perhaps, did the symptoms of chicken-pox so nearly resemble those of the small-pox, nor these diseases more fully assume the characters of each other.*" At the commencement of the epidemic, Professor B. regarded the two diseases as perfectly distinct, but running a common race; but at length he was inclined to consider them as identical; thus making small-pox and varicella the same disease, making the same mistake as was made by Professor Thomson, and is still made by many, from not knowing that in some

---

modified cases of variola, whether that modification be produced by vaccination or other causes, the eruption takes on a great variety of forms, simulating not only varicella, but also other eruptive diseases. Indeed, it would not be difficult to show, if we admit what has been contended for by Thomson, Berard, Willan, Ashby Smith, Frank, Heberden, and others, that small-pox, plague, chicken-pox, pemphigus, pompholyx, kine-pock, grease-pox, measles, and scarlet fever, are all modifications of the same disease, and derivable from one common virus.

During a dispensary practice of several years, I was in the constant habit of seeing what was called by myself, and others, chicken-pox, but which I am now satisfied was small-pox. In the same family, I have attended cases of confluent small-pox of the most malignant type, while others had what usually goes under the name of *varioloid*, and others still had aricella; that is, "an eruption over the body of semi-transparent glabose vesicles, with red margins accompanying a slight attack of fever, seldom passing into suppuration; but on the third day, bursting at their tips, concreting into small puckered scabs, and leaving no cicatrices."*

Precisely what Cazenave has observed with regard to certain eruptive diseases in Paris, "the same cause, viz., variolous infection, seemed to develop these several eruptions, which were observed in the same quarters, in the same streets, in the same houses. When the disease made its appearance among a numerous family, some had small-pox, some modified small-pox, and others chicken-pox. One circumstance was striking to every one, viz., the mildness of the disease in those persons who had been vaccinated, and in the majority of those who had already had variola." I cannot agree with Dr. Copland, in believing that such facts as these favor the opinion of Dr. Thomson, that chicken-pox and small-pox are the same disease; on the contrary, I am led to the conviction that, in one form of modified small-pox, the eruption is strictly vesicular, and has the above characters of variola, so as not to be distinguished from it by the character of the vesicles alone.

The following points, however, are to be observed:

1*st.* That true varicella is not transmitted by inoculation, and never produces variola; while the matter of small-pox always produces small-pox by inoculation. The matter of varicella, when inserted beneath the cuticle, may cause local irritation and redness, and in some cases, a vesicle, which may go through its ordinary stages, but without any, or slight constitutional disturbance.

2*d.* Where chicken-pox has been regarded as contagious, it has been confounded with modified small-pox.

3*d.* Varicelloid variola, as well as true varicella, may appear in persons who have not been vaccinated, as well as those that have been, although, as a general rule, the former occurs only in those who have been protected by kine-pock, or by a previous attack of small-pox.

4*th.* Vaccination will pursue its usual regular course when practiced shortly after an attack of true *varicella,* which never happens when vaccination follows true variola.

5*th.* The progress of true varicella is uniformly the same, whether it occurs before or after vaccination, or after variola.

---

* Copland, *Dict. of Med.*

*6th.* As true varicella may prevail as an epidemic without any admixture of varioloid eruptions, or variola, so may variola prevail without the varioloid variety; although, at certain seasons, and in some localities, from the operation of certain unknown causes, variola may give rise to a great variety of eruptions, simulating, in fact, a number of cutaneous diseases hitherto regarded as specifically distinct, as varicella, pemphigus, purpura, pompholyx, erysipelas, &c.

*7th.* The eruption in variolous cases occurs on the third day; in true varicella, on the second day.

*8th.* True varicella includes different varieties of eruptions, consisting of vesicles, or very imperfect pustules, which maturate and decline in three, four, or five days, characterized by slight antecedent fever, and occurring chiefly in infancy and childhood, occasionally in adult age, and at times as an epidemic.

*9th.* These varieties have been called small-pox, chicken-pox, and hives, corresponding to the forms described by Willan and Bateman. 1*st. Varicella lentiformis;* 2*d. V. coniformis;* 3*d. V. globularis;* to which may be added a fourth variety, the *V. corymbosa* of Gaad—the confluent chicken-pox.

*10th.* These varieties may readily be distinguished by the following characteristics, which I chiefly take from Copland.

In the first variety, (lenticular,) small red protuberances appear on the first day, of an irregularly circular form, with almost a flat and shining surface, in the center of which a transparent vesicle is soon formed; a whitish lymph fills the vesicle on the second day, which is about one-tenth of an inch in diameter; the lymph is straw-colored on the third day; and, on the fourth, the vesicles which have not been broken subside, and are puckered at their margins; few are entire on the fifth day; and, on the sixth, small brown scales appear in place of the vesicles, becoming yellowish on the seventh or eighth, gradually drying from the circumference to the center; falling off on the ninth and tenth days, leaving, for a time, red marks on the skin, without depression. The disease, however, may be protracted several days, owing to successive crops of vesicles making their appearance. The eruption appears *first on the back and breast*, is usually distinct, and general over the body. No cicatrices are left, even when the vesicles suppurate. (The eruption of variola appears first on the face, neck, and hands.)

In the second variety, (*conoidal,*) the vesicles are acuminated on the first day, and contain transparent lymph, having a somewhat hard and slightly inflamed base. On the second day, they are a little larger, bases more inflamed, and the lymph, in many of them, of a light straw color. The vesicles are shriveled on the third day, and the lymph of those broken is concreted into slight gummy scabs. Such as remain whole, and have their bases much inflamed, contain, on the third day, a whitish puriform fluid; *every vesicle of this kind leaving, after scabbing,* a durable cicatrix. On the fourth day, thin, dark-brown scabs are seen, intermixed with others which are rounded, yellowish, and semi-transparent, and then gradually dry, separate, and fall off in four or five days. The whole duration of the eruptive stage, in this variety of the disease, is six days, owing to a fresh eruption of vesicles usually taking place on the second or third day, which has a similar course to the preceding; minute red tubercles appear in some cases, and subside without forming vesicles. The scales last formed are generally not separated till the eleventh or twelfth day, when slight ulceration takes place in vesicles from which the scabs have fallen off, which is apt to take place where the febrile symptoms have been severe. Depressions or cicatrices may remain, but this only in parts subjected to pressure.

In the third variety, (*globular,*) the vesicles are large and globular, the base not quite circular; they are surrounded by inflammation, and contain a transparent lymph,which is slightly turbid, and resembles milk whey, on the second day of the eruption. They subside on the third day, and become shriveled, as in the former varieties, and appear yellowish from their being mixed with the lymph. Before the end of the fourth day, the cuticle separates, and thin, dark scabs cover the base of the vesicles. The scabs dry and fall off in four or five days afterward.

11*th.* The reader must have noticed in the above descriptions, some of the forms of varioloid eruptions, as they usually occur, particularly in the second and third varieties; but, as a general rule, the vesicle, full of serum at its apex on the first day of the eruption; their irregular form, and early abrasion; the shriveled state of those that remain entire on the third and fourth days, and the general appearance of small scabs on the fifth day, when small-pox pustules are not at the height of their suppuration, will usually enable the practitioner to form a correct diagnosis. I am satisfied, too, that the mark laid down by Willan is a very important one, namely, that variolous pustules are, on the first and second day, small, *hard,* globular, red, and painful, imparting the sensation, when the finger is passed over them, similar to that which one might conceive would be excited by the pressure of small, round seeds under the cuticle. In varicella, as Copland has remarked, almost every vesicle has, on the first day, a hard, inflamed *margin;* but the sensation communicated to the finger is like that from a round seed flattened by pressure. But I can by no means subscribe to the truth of the following remark, which immediately follows:—"When the globular vesicles, or hives, appear, as is sometimes the case, intermixed with the lenticular or conoidal eruption, they afford a ready distinction from the small-pox, to the pustules of which they have little resemblance." I have, however, seen both these forms of eruption proceed from variolous infection, not only in the epidemic as it prevailed in Gorham, but also in the city of New-York and other places.

12*th.* There can be no doubt that the precursory symptoms of modified small-pox are usually more intense than those of varicella, though there are some notable exceptions to this, as at Gorham, where many persons, attacked with the disease, retained their appetite for food, and their usual strength, keeping about their ordinary business, and saying they never felt better, even when the body was sprinkled with a considerable number of well-developed pock; while I have often known true varicella preceded and attended by considerable depression, anorexia, and chills, followed by high fever, pain in the head and back, thirst, and other constitutional symptoms.

13*th.* Dr. Copland also states that, in modified variola, the eruption is *pustulent,* and the pustules are small, circular, and generally depressed in the center; and, after the scaly crusts drop off, tubercles are frequently seen, which disappear but slowly; while, in varicella, the vesicles, which are at first transparent, contain a fluid which becomes sero-purulent; and they are never followed by tubercles, as in modified variola. We have seen that there are exceptions to the distinctions here pointed out, though they will doubtless generally hold. All will agree with him in the opinion that varicella is not infectious.

14*th*. There is one mark of true varicella, quite characteristic, but not often pointed out, which relates to the form, &c., of the crusts. In varicella these are irregular, uneven, opaque, of a pale brownish or yellowish color, formed of lymph and collapsed cuticle, and falling off, not in a single piece like the crusts of variola, but in small fragments.* Professor Thomson, it seems, never saw chicken-pox prevailing, unless intermingled with small-pox at the same time; while nothing is more common, at the present day, than to see chicken-pox prevailing as an epidemic, without any cases of small-pox whatever. This, were there no other test, would be ample, as diagnostic of true varicella.

15*th*. Again, while an attack of natural small-pox protects the system, as a general rule, against a recurrence of small-pox, and an attack of chicken-pox against a recurrence of that disease, neither of these affords the slightest security against the other. This proves the intrinsic qualities of each virus to be distinct.

16*th*. I have now known four instances where an eruptive disease, appearing in different parts of the country, has been pronounced, by experienced physicians, chicken-pox; but which, after the disease had extended, and the virus diffused far and wide, proved to be small-pox, several cases terminating fatally. This shows the necessity of physicians being on their guard, and of making themselves thoroughly acquainted with the real characteristics of the disease, if there are any, so that such mistakes may be avoided.

17*th*. Where doubt exists, we should go on the supposition that the disease is variola, subjecting those exposed, to vaccination, and taking all necessary precautions for preventing the spread of the infection.—*N. Y. Jour. of Medicine.*

* *Ed. Med. and Surg. Jour.*, Ap. 1820, and Jan. 1828.

